# Evidence of niche shift and global invasion potential of the Tawny Crazy ant, *Nylanderia fulva*

**DOI:** 10.1002/ece3.1737

**Published:** 2015-09-30

**Authors:** Sunil Kumar, Edward G. LeBrun, Thomas J. Stohlgren, Jared A. Stabach, Danny L. McDonald, David H. Oi, John S. LaPolla

**Affiliations:** ^1^ Natural Resource Ecology LaboratoryColorado State UniversityFort CollinsColorado80523‐1499; ^2^ Department of Ecosystem Science and SustainabilityColorado State UniversityFort CollinsColorado80523‐1499; ^3^ Brackenridge Field LaboratorySection of Integrative BiologyUniversity of Texas at Austin2907 Lake Austin BlvdAustinTexas78703; ^4^ Texas Research Institute for Environmental StudiesSam Houston State UniversityHuntsvilleTexas77341‐2506; ^5^ USDA‐ARSCenter for Medical, Agricultural & Veterinary EntomologyGainesvilleFlorida32608‐1067; ^6^ Department of Biological SciencesTowson UniversityTowsonMaryland21252‐0001

**Keywords:** Biological invasions, biotic homogenization, ecological niche models, invasion stages, MaxEnt, niche expansion, risk analysis, species distribution modeling

## Abstract

Analysis of an invasive species' niche shift between native and introduced ranges, along with potential distribution maps, can provide valuable information about its invasive potential. The tawny crazy ant, *Nylanderia fulva*, is a rapidly emerging and economically important invasive species in the southern United States. It is originally from east‐central South America and has also invaded Colombia and the Caribbean Islands. Our objectives were to generate a global potential distribution map for *N. fulva*, identify important climatic drivers associated with its current distribution, and test whether *N. fulva*'s realized climatic niche has shifted across its invasive range. We used MaxEnt niche model to map the potential distribution of *N. fulva* using its native and invaded range occurrences and climatic variables. We used principal component analysis methods for investigating potential shifts in the realized climatic niche of *N. fulva* during invasion. We found strong evidence for a shift in the realized climatic niche of *N. fulva* across its invasive range. Our models predicted potentially suitable habitat for *N. fulva* in the United States and other parts of the world. Our analyses suggest that the majority of observed occurrences of *N. fulva* in the United States represent stabilizing populations. Mean diurnal range in temperature, degree days at ≥10°C, and precipitation of driest quarter were the most important variables associated with *N. fulva* distribution. The climatic niche expansion demonstrated in our study may suggest significant plasticity in the ability of *N. fulva* to survive in areas with diverse temperature ranges shown by its tolerance for environmental conditions in the southern United States, Caribbean Islands, and Colombia. The risk maps produced in this study can be useful in preventing *N. fulva's* future spread, and in managing and monitoring currently infested areas.

## Introduction

Rapidly increasing global trade and human movement have accelerated the rate of species introductions and establishment into novel areas across the world (Mack et al. 2000). Invasive species can negatively affect native ecosystems, agriculture, forestry, animal, and human health and cause enormous economic losses (Pimentel et al. 2005). They can also cause local extinction of rare and unique native species resulting in biotic homogenization and are considered as one of the greatest threats to biodiversity worldwide (Sax et al. 2002). Maps of species' current and potential distributions are valuable tools for resource managers for preventing the introduction or establishment of invasive alien species, and for designing an effective early detection and rapid response system (Peterson 2003; Jimenez‐Valverde et al. 2011). Non‐native species, when introduced to new geographic areas, may establish in environmental conditions different from their native range because of absence of natural enemies or local adaptation. Therefore, analysis of how a species' niche may have changed between native and introduced ranges may be useful in understanding range expansion and invasion potential (González‐Moreno et al. 2015).

**Figure 1:**
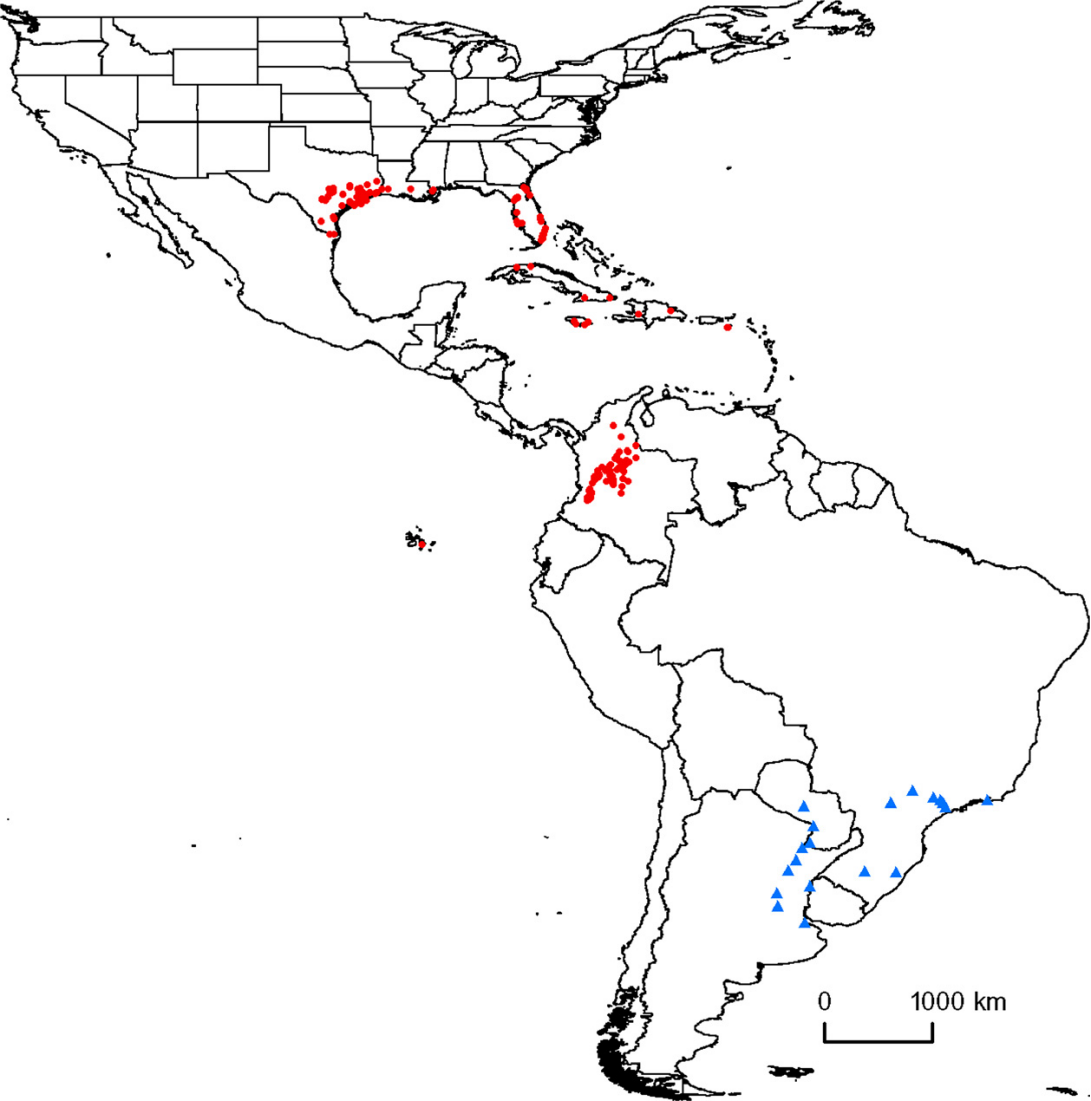
Known native (blue triangles) and invasive (red circles) occurrences of *Nylanderia fulva* in South America, Caribbean islands, and southern United States.

Several invasive ant species around the world have caused economic losses, affected human health, decreased agricultural production, damaged infrastructure, and reduced the diversity of local ant and arthropod assemblages (Holway et al. 2002a; Gutrich et al. 2007). An alien ant species, the tawny crazy ant, *Nylanderia fulva* (Mayr) (Hymenoptera: Formicidae), previously *Paratrechina fulva* (LaPolla et al. 2010), and originally referred to as *Paratrechina* nr. *pubens*, is invading the southern United States (Gotzek et al. 2012). Its occurrence in the United States (US) was first documented in Houston in 2002 (Meyers and Gold 2008) and may have arrived in Florida earlier (Klotz et al. 1995; Deyrup et al. 2000), but collections of the very similar *P. pubens* from Florida dating back to the 1950s (Trager 1984) prevent an accurate identification of early pest populations. At present, populations of this species occur in 27 counties in Texas, 27 counties in Florida, and several counties in southern Mississippi (MacGown and Layton 2010) and southern Louisiana (Hooper‐Bui et al. 2010; Fig. 1). Introduction of *N. fulva* in Colombia caused extensive ecological and agricultural damage (Zenner de Polania 1990). In the Southern United States, *N. fulva* displaces red imported fire ants (*Solenopsis invicta*), and regionally distributed native species, thereby reducing both biological and functional diversity (LeBrun et al. 2013). *Nylanderia fulva* can also transport pathogens of plants, humans, and other animals (McDonald 2012). Nests within populations contain multiple queens (Zenner de Polania 1990). Interconnected nests of these ants form extraordinarily dense populations that greatly exceed the combined densities of all ants in adjacent uninvaded assemblages (LeBrun et al. 2013). They feed on small insects and vertebrates, and honeydew secreted by aphids (Zenner de Polania and Bolaños 1985). They invade people's homes, nest in crawl spaces and walls, and damage electrical equipment resulting in millions of dollars of losses (Blackwell 2014). Populations spread about 200 m per year as a result of nest fission at the invasion front (Meyers and Gold 2008). Female reproductives of *N. fulva* have not been observed to engage in alate flights, so long‐distance dispersal occurs largely as a result of human transport of nesting ants. Despite *N. fulva* being a potentially devastating invasive species, no information currently exists on its potential distribution in the United States. There is an acute need for climate‐based projection of the invasion potential of *N. fulva* in the near‐term to guide conservation (e.g., potential biocontrol; Waltari and Perkins 2010).

Availability of suitable environmental conditions is a prerequisite for population establishment. Species environment matching models, also called habitat models, ecological niche models (ENM), and species distribution models (SDM), quantify the range of environmental conditions to assure viable populations. These models are based on the “niche” concept (hereafter niche models), which can be defined as the multivariate environmental space within which a species can live indefinitely without immigrational subsidy (Grinnell 1917; Hutchinson 1957). The fundamental niche represents the conditions where a species *can* live indefinitely, whereas a species' realized niche is where a species actually lives; species do not occupy all portions of their fundamental niche because of biotic constraints (e.g., competition or lack of host species) or dispersal limitations (Peterson et al. 2011). Niche models can be broadly classified as correlative models or process‐based/mechanistic models (Dormann et al. 2012). The correlative niche models map the realized or potential distribution of a species by associating occurrence data with environmental data (Jimenez‐Valverde et al. 2011) and are widely used tools for assessing the risk of invasive species (e.g., Peterson 2003; Kumar et al. 2009, 2014; Menke et al. 2009; Roura‐Pascual et al. 2009; Stohlgren et al. 2010).

Numerous correlative niche modeling techniques are available for mapping the risk of invasive species (Peterson et al. 2011). These can be broadly categorized into presence‐only (e.g., BIOCLIM, DOMAIN), presence‐background (e.g., MaxEnt, GARP), and presence–absence (GLM, GAM, CART) models. Presence‐only and presence‐background (i.e., randomly selected absences from areas that have been accessible to the species) niche models are better suited for modeling potential distributions of invasive species because absence data for such species may not be reliable; a species may go undetected or it may not have had enough time to disperse to new locations yet (Jimenez‐Valverde et al. 2011). The presence–absence models are more suitable for estimating actual distributions of a species.

The use of presence‐only or presence‐background correlative niche models for mapping the potential distributions of introduced invasive species can be challenging because the species may not yet have reached equilibrium within its invaded environment (Vaclavik and Meentemeyer 2009). Therefore, a model trained only with invaded range occurrences may highly underestimate areas where a species may potentially exist (Jimenez‐Valverde et al. 2008), thus providing inaccurate information for management actions and policy development. This problem can be overcome by developing a model using species occurrence data from native *and* invaded ranges (e.g., Fitzpatrick et al. 2007; Broennimann and Guisan 2008). The presence‐only data, if not collected using statistically designed field surveys, may also have locational and taxonomic uncertainties, environmental and spatial bias, and may come from sink populations (Wolmarans et al. 2010). Therefore, care must be taken in using data from a variety of sources, and models must be corrected for potential biases for accurate predictions.

Despite the high economic and ecological importance of *N. fulva*, very little information currently exists on its global distribution or the potential environmental factors that constrain its distribution. Land managers urgently need such information for managing currently infested areas and planning for prevention of future invasions. Our objectives were to: (1) map the global potential distribution of *N. fulva*; (2) identify climatic drivers associated with *N. fulva* distribution; (3) test whether the climatic niche of *N. fulva* has shifted during invasion; and (4) make inferences about the invasion stages of *N. fulva* in the United States.

## Methods

### Species occurrence data

Occurrence data for *N. fulva* were compiled from specimens at natural history museums, personal collections, scientific literature, and field surveys (Fig. 1; see Table S1 in Appendix S1 in Supporting Information). Taxonomic details on how we defined native and invasive ranges of *N. fulva*, and acceptance criteria for occurrence records are provided in Appendix S1.

Twenty‐seven presence records were collected from *N. fulva*'s native range including Argentina, Brazil, and Paraguay (Fig. 1; Table S1 in Appendix S1). We collected 311 records from the invaded range outside the continental United States (CONUS), and 1061 records from within CONUS (Florida, Louisiana, Mississippi, and Texas). Colombian data were provided by A. Arcila. Thus, a total of 1399 occurrence records covering South America, Caribbean islands, and southern United States were available for modeling. The total number of presence records was reduced to 307 after removing duplicate data points (i.e., more than 1 point within a ~1 km^2^ grid cell) and applying spatial filtering to reduce the effect of spatial autocorrelation on models (Boria et al. 2014).

### Environmental data

A total of 20 bioclimatic variables were considered in developing *N. fulva* potential distribution models (Table S2 in Appendix S1). These included 19 bioclim variables from the WorldClim dataset at ~1‐km resolution (Hijmans et al. 2005). These bioclim variables were derived using monthly temperature and precipitation data covering a period from ~1950 to 2000, and represent average temperature and precipitation, seasonal variables, and climatic extreme indices (Hijmans et al. 2005). They are considered biologically meaningful as they aggregate climate information that influences biological processes. Additionally, “degree days with average temperature ≥10°C” variable was generated in Arc Map (ESRI, Redlands, CA) using average monthly temperature data based on *N. fulva'*s responses to different temperatures (Arcila et al. 2002a,b; McDonald 2012). These variables were chosen based on our knowledge of *N. fulva* ecology (McDonald 2012; LeBrun et al. 2013), and their use in previous invasive ant species modeling studies (Menke et al. 2009; Roura‐Pascual et al. 2009). Highly collinear variables (Pearson correlation coefficient, ¦r¦ ≥ 0.80) were removed, and only one variable from a set of highly correlated variables was included in the same model (Table S3 in Appendix S1). All geographical information system (GIS) layers were projected to an equal area projection (World Cylindrical Equal Area Conic projection, Datum WGS1984).

### Model calibration and validation

Maximum entropy model, MaxEnt (version 3.3.3k; Phillips et al. 2006), was used for mapping potential distribution of *N. fulva*. The MaxEnt model was chosen because (1) it uses presence‐background data; species true absences are not required; (2) generally performs better than other niche modeling algorithms (Evangelista et al. 2008; Kumar et al. 2009); and (3) is relatively robust to small sample sizes (Guisan et al. 2007a,b; Kumar and Stohlgren 2009). MaxEnt uses species occurrence data and spatial environmental variables and produces an index of relative suitability that varies from 0 (unsuitable or most dissimilar to presence locations) to 1 (most suitable or most similar to presence locations). Background points (50,000 for the MaxEnt model) were randomly selected from areas that have been accessible to *N. fulva* using the “Biotic‐Abiotic‐Mobility” (BAM) framework (Soberon and Peterson 2005). We suspected a sampling bias because the occurrence data were not collected randomly and came from multiple sources. Thus, we generated a bias surface using a kernel density estimate using SDMToolbox (Brown 2014). The bias surface was used in MaxEnt to weight the selection of background points to account for sampling intensity and potential sampling bias (Elith et al. 2010; Syfert et al. 2013). Three models were fitted: (1) invasive range model calibrated using only the continental US occurrence data (IRM‐CONUS); (2) native and invasive range model calibrated using occurrence data from the Americas (NIRM‐Americas); and (3) all occurrence data with the global background (NIRM‐Global; Table 1). Background points for NIRM‐Global model were randomly drawn from all terrestrial areas of the world assuming unlimited dispersal (Table 1).

**Table 1:** Areas of calibration and performance statistics for different models

Model	Area of calibration/background extent	MaxEnt settings	Test AUC_cv_	pAUC	Test sensitivity	
					0% OR	10% OR
IRM‐CONUS	Continental United States of America (USA)	Linear, Quadratic and Hinge features; *β *= 1.5	0.961 (±0.01)	1.96 (±0.01)	0.006	0.110
NIRM‐Americas	Continental USA, Caribbean islands, and South America	Linear, Quadratic and Product features; *β *= 2.5	0.937 (±0.02)	1.82 (±0.06)	0.006	0.114
NIRM‐Global	Global (all terrestrial areas of the world)	Linear, Quadratic, Product and Hinge features; *β *= 2.5	0.966 (±0.01)	1.91 (±0.03)	0.003	0.105

Note: *β* is regularization multiplier; OR is training omission rate. Test AUC_cv_ is MaxEnt generated 10‐fold cross‐validation area under the ROC curve; pAUC is partial AUC ratio calculated at 0% omission rate (Peterson et al. 2008). The AUC_cv_ and pAUC values are not comparable across models because models were calibrated at different extents.

As default settings in MaxEnt do not always produce the best predictions (Merow et al. 2013; Kumar et al. 2014), it was run with combinations of different feature types and regularization multiplier values (ranging from 1 to 3). The ENMTools (Warren et al. 2010) was used to calculate Akaike's information criterion (AIC) values for MaxEnt models with different settings at different extents of calibration, and models with optimal complexity were retained for further evaluation (Table S4 in Appendix S2). The 10‐fold cross‐validation was used in MaxEnt, and the area under the ROC (receiver‐operating characteristic) curve (AUC_cv_; Fielding and Bell 1997) values were reported. In addition, the partial area under the ROC curve (pAUC) ratio was used for evaluating model performance (Peterson et al. 2008). The pAUC ratio values were calculated using a Visual Basic program (Barve 2008). A pAUC ratio >1.0 indicates better than random model performance. The sensitivity index (i.e., number of correctly classified presences) was also used as an additional metric to evaluate model performance. Test sensitivity was calculated at 0% and 10% training omission rates (see Liu et al. 2013; Kumar et al. 2014). The best models for each extent of calibration were selected based on AIC, AUC_cv_, pAUC values, and omission rates. In addition, the response curves generated by MaxEnt were evaluated for their biological relevance to *N. fulva*, and models that resulted in biologically nonsensical (i.e., highly jagged or multimodal) curves were eliminated or ranked low (Table S4 in Appendix S2).

### Realized niche shift and invasion stage analyses

The principal component analysis (PCA) approach proposed by Broennimann et al. (2012) was used to test any potential niche shift by quantifying climatic niche space for *N. fulva* at different extents. This method compares the environmental conditions available for a species within a defined study extent (background) with its observed occurrences and calculates the available environmental space defined by the first two axes from the PCA. The same 20 climatic variables, as used in MaxEnt, were used for the PCA. This method automatically corrects for sampling bias using a smooth kernel density function (Broennimann et al. 2012). The niche overlap score for each comparison was calculated using Schoener's *D* index (Schoener 1970), which varies from 0 (no overlap between niches) to 1 (complete overlap). The statistical significance of niche overlap index (*D*) was tested against chance using 100 randomizations (alpha = 0.05). The R code for the PCA was modified from Broennimann et al. (2012). We compared native and invasive niche spaces for *N. fulva* using three regional contrasts: (1) native vs. invasive_CONUS (invasive occurrences from the continental US); (2) native vs. invasive_Non‐CONUS (invasive occurrences from outside CONUS); (3) invasive_CONUS vs. invasive_Non‐CONUS; and (4) native vs. invasive (all invasive range occurrences).

We adopted a theoretical framework suggested by Gallien et al. (2012) to identify stages of *N. fulva* invasion in the continental United States by plotting predicted probabilities from the “invasive_CONUS” model (regional) against the native and invasive occurrences combined model (NIRM‐Americas). This framework helps in making inferences about the stages of invasion for different populations of a species in the ecological niche space (Gallien et al. 2012). In the niche space, a species would be at quasi‐equilibrium if the regional and global models predict higher probabilities (e.g., >0.5) for the species' presence (stabilizing populations); regional colonization occurs when the regional model predicts low probability of presence, but the global model predicts high probability. However, if the regional and global models predict low probability of presence, these occurrences may represent population sinks. In contrast, evidence that some form of regional adaptation may be occurring arises when the regional model predicts high probabilities of presence for some set of occurrences, but the global model predicts low probabilities (Gallien et al. 2012).

All GIS analyses were performed using ArcGIS version 10.2.2 (ESRI). All statistical analyses were conducted in R (R Development Core Team, 2013).

## Results

### Model performance and variable importance

All three models (IRM‐CONUS, NIRM‐Americas, and NIRM‐Global) performed better than random with AUC_cv_ values ranging from 0.94 to 0.97, and pAUC values from 1.82 to 1.96 (Table 1). The models had low omission rates; test sensitivity at 0% training omission rate varied from 0.003 to 0.006, and at 10% training omission rate varied from 0.105 to 0.114 (Table 1). The best model for the continental United States (IRM‐CONUS) included four climatic variables, whereas NIRM‐Americas and NIRM‐Global models each included six variables (Table 2). The best IRM‐CONUS model included Linear, Quadratic, and Hinge features (regularization multiplier [RM] = 1.5), whereas the NIRM‐Americas model included Linear, Quadratic, and Product features (RM = 2.5). The NIRM‐Global model included Linear, Quadratic, Product and Hinge features (RM = 2.5; Table 1). The NIRM‐Global model with moderate level of complexity ranked highest compared to other models with lower or higher levels of complexity (Table S4 in Appendix S2).

**Table 2:** Average percent contribution of environmental variables to different *Nylanderia fulva* models; values were averaged across 10 replicate runs

Variable	IRM‐CONUS	NIRM‐ Americas	NIRM‐Global
Degree days with average temp. ≥10°C (degdays10)^a^	90.1	25.3	24.5
Precipitation of driest quarter (bio17; mm)	6.5	5.2	65.0
Mean temperature of wettest quarter (bio8; °C)^a^	1.9	–	–
Temperature seasonality (SD × 100) (bio4)^b^	1.4	17.8	–
Mean diurnal range in temp. (bio2; °C)	–	26.2	0.4
Precipitation seasonality (CV) (bio15)	–	16.8	3.1
Precipitation of wettest quarter (bio16; mm)	–	8.6	2.6
Isothermality (bio3)^b^	–	–	4.5

Note: IRM‐CONUS is the invasive range model using occurrence data from only continental United States; NIRM‐America is the native and invasive range model using data from Americas; NIRM‐Global is the native and invasive range model using data from Americas but calibrated using global extent background data.

a Variables highly correlated at NIRM‐Americas and NIRM‐Global extents (Pearson's correlation coefficient |r| ≥ 0.80).

b Variables highly correlated at all three extents.

The mean diurnal range in temperature, degree days with average temperature ≥10°C, and precipitation of driest quarter were the most important climatic variables associated with *N. fulva* distribution (Table 2). The importance of variables slightly changed with the calibration extent (Table 2). For example, degree days at ≥10°C was the top most important predictor in the IRM‐CONUS model, but it ranked second in NIRM‐Americas and NIRM‐Global models (Table 2). The jackknife test of variable importance showed that the degree days at ≥10°C had the most information that was not present in other variables (NIRM‐Americas model; Figure S1 in Appendix S3). The probability of *N. fulva* presence was highest when mean diurnal range in temperature was between 6 to 11°C, and degree days at ≥10°C was between 3000 and 5000 (Figure S2A, B in Appendix S3). The probability of *N. fulva* presence was higher at lower levels of temperature and precipitation seasonality (Figure S2C, D in Appendix S3).

### Predicted potential distribution of *Nylanderia fulva* in the United States

**Figure 2:**
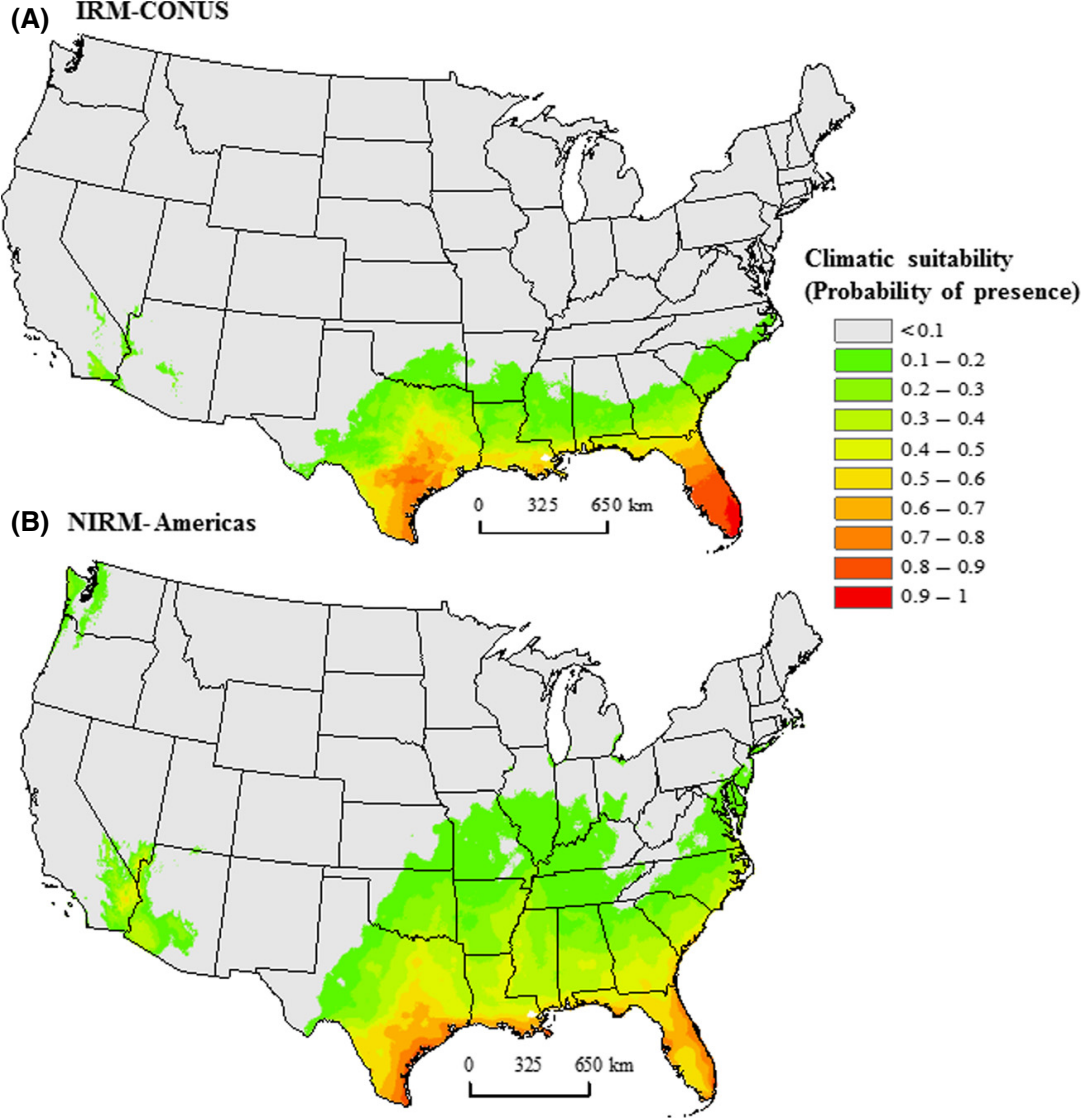
Predicted potential distribution of *Nylanderia fulva* in the continental United States based on occurrences from (A) invaded range in southern United States (IRM‐CONUS), and (B) native and invaded range combined (NIRM‐Americas).

**Figure 3:**
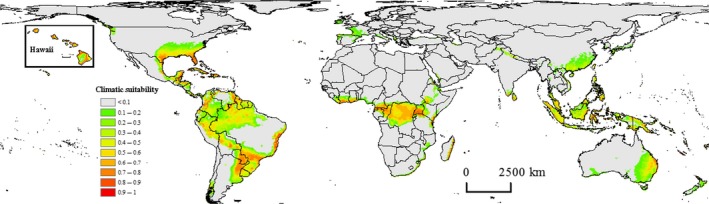
Global potential distribution of *Nylanderia fulva* based on native and invasive range global (NIRM‐Global) model.

The predicted potential distribution of *N. fulva* closely matched observed occurrences (Figs. 2 and 3 vs. Fig. 1). Both IRM‐CONUS and NIRM‐Americas models predicted highly suitable areas for *N. fulva* in southeastern Texas, southern Mississippi, southern Louisiana, and most of Florida (Fig. 2). The NIRM‐Americas model predicted low‐to‐medium suitability in southeastern parts of California, southern Nevada, and southwestern Arizona, whereas the IRM‐CONUS model predicted very low suitability in these areas (Fig. 2). The NIRM‐Americas and NIRM‐Global models predicted low suitability for *N. fulva* in northwestern Washington and northern Oregon (Figs. 2 and 3). The partial model using *N. fulva* invaded range occurrences from the southern United States (IRM‐CONUS; Fig. 2A) predicted less expansive regions of the suitable habitat compared to a full model using all native and invaded range occurrences (NIRM‐Americas; Fig. 2B). The full model predicted lower climatic suitability for *N. fulva* as far north as southern Missouri, Illinois, and Indiana (Fig. 2B). However, both models largely agree on areas of high probability (>0.5) of suitable conditions, these were largely restricted to the Gulf and Southern Atlantic Coast regions, plus coastal and Central Texas. The NIRM‐Global model predicted highly suitable areas for *N. fulva* in all the Hawaiian Islands (see inset in Fig. 3).

### Global potential distribution of *Nylanderia fulva*

The NIRM‐Global model predicted highly suitable areas for *N. fulva* in eastern Mexico, the Caribbean islands, and Central America (Fig. 3). The model predicted highly suitable areas in western Colombia, western, southern and eastern coastal Brazil, Ecuador (including the Galapagos Islands), northern Peru, northern Bolivia, eastern Paraguay, Uruguay, and western Argentina (Fig. 3). The model predicted suitable areas in central parts of Africa, eastern Madagascar, lower Himalayas in India and Pakistan, southern India and Sri Lanka, southeastern China (including Taiwan), southeastern parts of Asia, eastern Australia, and northern parts of New Zealand (Fig. 3). Based on observed presences in the Americas (Fig. 1), *N. fulva* currently occurs in areas with an average annual temperature between 13 and 29°C, and an average annual precipitation between 378 and 4900 mm (Table S2 in Appendix S1).

### Niche shift and stages of invasion

**Figure 4:**
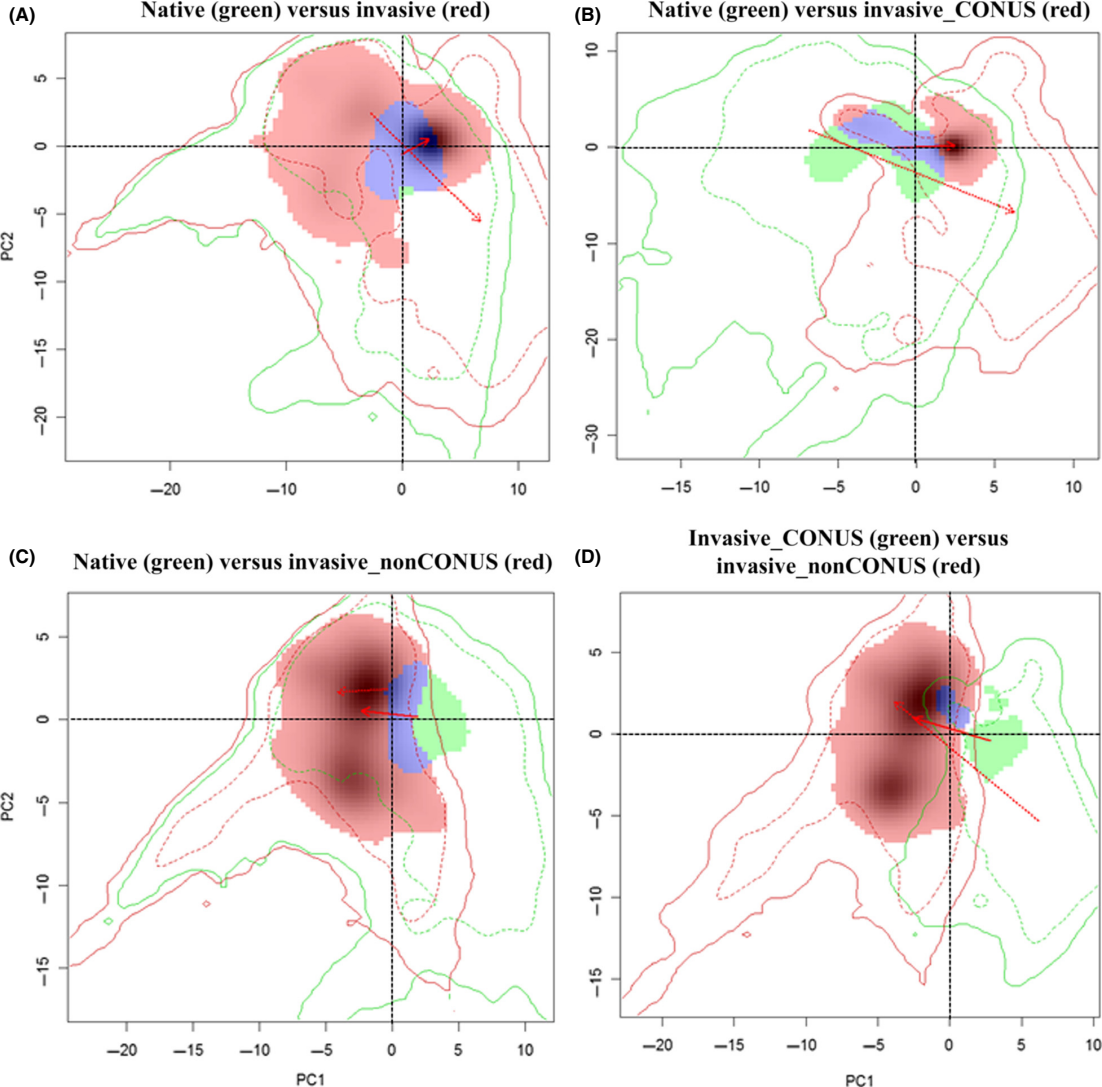
Native and invasive niches of *Nylanderia fulva* in different regions; multivariate climatic space was calculated using PCA‐env method. PC1 and PC2 represent the first two axes of the principal competent analysis (PCA). The green and red shadings represent density of species occurrences in different regions; blue represents overlap. Solid and dashed lines show 100% and 50% of the available (background) environment. The red arrows show how the center of the climatic niche for *N. fulva* (solid) and background extent (dotted) has moved between two ranges.

The principal component analysis (PCA) showed that the realized climatic niche of *N. fulva* may have shifted and expanded in the invasive regions examined; the center of the realized climatic niche moved toward warmer temperatures and higher temperature seasonality, and there was only 24% niche overlap between native and invaded ranges (Schoener's *D *=* *0.24; Fig. 4A). The PCA showed a similar realized niche shift and expansion in the southern United States occurrences with only 24% niche overlap (Fig. 4B); the climate space in the United States that is currently “unfilled” (green shaded areas within solid red contour line) by *N. fulva*. This may be representing geographic areas where *N. fulva* is currently undetected or absent. The amount of niche overlap was lower between *N. fulva*'s native and invaded ranges outside the continental United States (Schoener's *D *=* *0.16; Fig. 4C), although the niche expansion was higher (i.e., red shaded areas within solid red contour line; Fig. 4C). There was little niche overlap (8%) between *N. fulva*'s invaded ranges inside or outside the CONUS (Fig. 4D) suggesting a more extreme shift in the realized niche during invasion of the continental United States compared to Colombia and the Caribbean islands.

**Figure 5:**
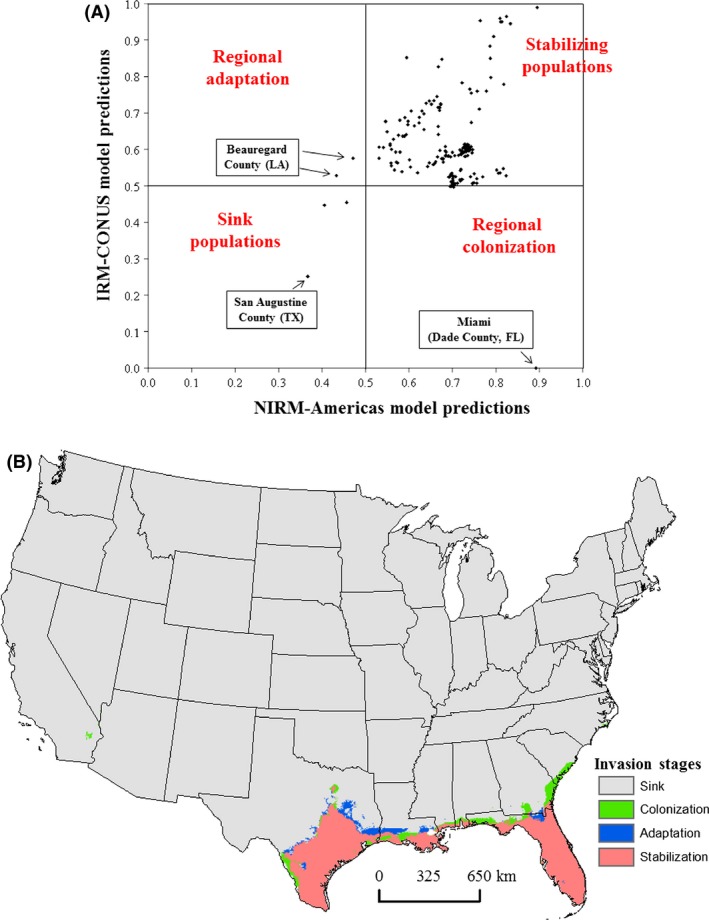
(A) *Nylanderia fulva* observed occurrences at different stages of invasion based on global and regional model predictions, and (B) mapped areas showing potential (hypothesized) for population stabilization, adaptation, colonization, and sink.

The analysis of current stages of *N. fulva* invasion in the United States based on regional (IRM‐CONUS) and global model (NIRM‐Americas) predictions revealed that the majority of observed *N. fulva* occurrences are stabilizing populations; one population (Miami, Dade County, Florida) may be at colonization stage, two at regional adaptation, and three represent sink populations (Fig. 5A). The majority of southern occurrences are in the stabilizing zone, whereas colonization and adaptation zones were predicted for northern populations toward the leading edge of *N. fulva* invasion (Fig. 5B).

## Discussion

Our study provides strong evidence of a shift in the realized climatic niche of *N. fulva* during its spread through Colombia, Caribbean islands, and southern United States. We showed that a partial model using invaded range occurrences from southern United States underestimated the potential risk of *N. fulva* invasion in the United States. Our models predicted potentially suitable habitat for *N. fulva* in southern parts of the United States, Hawaii, and other parts of the world. The predicted potential distribution of *N. fulva* conforms well to currently known occurrences in the southern United States and southeastern South America (Figs. 1 and 3), indicating that it meets biological expectations. The model predicted large amounts of climatically suitable areas in South America outside of the native range region occupied by this species. The absence of *N. fulva* from these areas suggests the existence of currently unappreciated biological constraints on its realized niche, potentially in the form of closely allied competitors or natural enemies. However, it may change with time with the arrival of new propagules through human transportation.

By applying the Gallien et al. (2012) theoretical framework, we found that the majority of observed occurrences of *N. fulva* in the United States are likely stabilizing populations. We quantified unfilled climatic niche space for *N. fulva* in the United States, where range expansion and colonization could occur. Indeed, new reports of *N. fulva* in Albany, Georgia (Dowdy 2013) and Mobile, Alabama (Carroll 2014) fall within this expansion zone. We identified the mean diurnal range in temperature, the number of degree days at ≥10°C, and precipitation of driest quarter as the most important variables associated with *N. fulva* distribution.

### Climatic niche shift

The niche shift and expansion quantified here shows *N. fulva*'s potential to invade novel areas. Our models indicated that the realized niche of *N. fulva* has shifted to warmer and drier conditions between the native and invasive ranges (Fig. 4A), likely due to a release from interspecific competition across the invasive range. *Nylanderia fulva* seems to occupy the majority of the climate space in its introduced range, matching its native range climatic space (Fig. 4A). However, the same results were not observed across the continental United States (Fig. 4B). This could be because *N. fulva*'s realized climatic niches in its invaded range are different within and outside the continental United States (Fig. 4D); the magnitude of the shift in realized niche was greater during *N. fulva* invasion into Colombia and the Caribbean Islands compared to continental United States (Fig. 4D).

The niche shift showed here is not unique to *N. fulva*. Several studies have documented climate niche shifts for other invasive ants and plant species (e.g., Broennimann et al., 2007; Fitzpatrick et al. 2007; Petitpierre et al., 2012; Guisan et al. 2014). Positive species interactions can expand the fundamental niche and range of a species, especially when species experience physical and biological stresses (He and Bertness 2014). Local adaptation of an introduced species in new geographic areas can occur because of its ability to exploit empty niches, or the frequency and magnitude of local disturbances creating new habitats, and the absence of its natural enemies (Sax et al. 2005).

Is the climatic niche of *N. fulva* evolving rapidly over time? We do not know. The issue of rate of niche evolution is a subject of ongoing debate; several studies suggest rapid niche evolution for some species (e.g., Holt and Gaines 1992; Sexton et al. 2009; Guisan et al. 2014), whereas other studies show niche conservatism over time (e.g., Peterson et al. 1999; Peterson 2011). Further research is needed to understand the changes and rates of *N. fulva*'s fundamental niche shifts.

### Caveats and uncertainties

Results from correlative niche models such as MaxEnt should be interpreted with caution because of inherent uncertainties and model specific assumptions. Niche model predictions may be affected by sampling bias, number of samples, incomplete species occurrence data, failure to account for biotic processes (e.g., presence of natural enemies), choice and spatial resolution of abiotic variables, multicollinearity, and species characteristics (Guisan et al. 2007a,b; Dormann et al., 2013; Syfert et al. 2013). A mismatch between the time period of species occurrences and climatic data might also influence niche estimates. Our models also do not account for microclimates available to the species because of the coarser spatial resolution (~1 km^2^) of climate dataset used in model calibration. Finer‐scale climate data (e.g., at a scale of a few square meters) were not available at the global level; the generation of such a dataset was beyond the scope of this study and would be impractical (Bennie et al. 2014). Thus, our results are applicable to population‐level responses of *N. fulva* to macroclimate rather than individual responses. Our models may have overestimated the potential suitable areas because not all predicted areas have suitable habitat for *N. fulva* (e.g., water bodies). Additionally, occurrences in urban areas where the species may be buffered from the natural climate envelope due to human habitat alterations (e.g., irrigation and structures) may cause the models to over predict suitability in nonurbanized areas.

*Nylanderia fulva* is a member of a taxonomically difficult group. Because of similarity in the worker caste among members of this genus, misidentifications in museum collections, and the literature occur (Gotzek et al. 2012). Due to the co‐occurrence of the morphologically similar, closely related species, *N. pubens*, in the Caribbean region records re‐reported herein from that region should be viewed as provisional and in need of additional collection to verify. Examination of the climatic values associated with these records from the Caribbean indicates that they cover conditions from within the climatic envelope for *N. fulva* (Appendix S1). Whether *N. fulva* as defined represents a single coherent biological entity has been questioned (Trager 1984; Kallal and Lapolla 2012). Additional studies of species boundaries within this group and population genetic studies of the source localities for invasive populations are needed because introduction history might determine the genetic diversity and structure of a species in invaded range (Ascunce et al. 2011; Le Roux et al. 2011); subspecies may have distinct climatic niches (e.g., Thompson et al. 2011).

The effects of environmental variables on species distributions are scale‐dependent. Environmental factors such as climate are generally associated with species' distributions at regional or continental scales, whereas biotic factors such as presence of a competitor or a host plant species control species distributions at local scale (Austin 2002). At the local scale, factors such as soil moisture and temperature can influence ant distribution and abundance (e.g., Holway et al. 2002b; Menke et al. 2009). Roura‐Pascual et al. (2004) found that the distribution of Argentine ants (*Linepithema humile* Mayr) may be limited by cooler temperatures and decreased humidity levels in the northern latitudes. The introduction and establishment of an alien insect species can also be affected by its behavior (e.g., nesting biology and social organization) and life history traits (Holway and Suarez 1999). Little is known about the abiotic, biotic, and phenological constraints that limit *N. fulva* distribution in its native and invasive range; our study generated hypotheses about these unknown factors, which could be experimentally tested. For example, our study showed that *N. fulva* is highly influenced by degree days at ≥10°C, and does quite well between 3000 and 5000 degree days (Figures S1 and S2 in Appendix S3). This finding can be tested in the laboratory.

Fine‐scale environmental heterogeneity may affect distribution and abundance of ant species (Savage et al. 2014), a factor not considered in our models because of the focus on climatic niche of *N. fulva*. Incorporating variables representing the fine‐scale heterogeneity at a finer spatial resolution might improve local and regional models. For example, remotely sensed indices such as Normalized Difference Vegetation Index (NDVI), and Enhanced Vegetation Index (EVI), soil moisture, and anthropogenic factors (e.g., Human Footprint Index) could be used to develop finer resolution local or regional models of *N. fulva* distribution. Future research should also investigate *N. fulva*'s response to climate change. Given the strong association of climatic factors with the distribution of *N. fulva*, it is highly likely that its future distribution may be affected by climate change.

The eradication of *N. fulva* from its invasive range appears to be an unachievable goal given its current levels of infestation. Therefore, prevention and control would be a better strategy for managing *N. fulva* invasion (Hoffmann et al. 2010). Prioritizing the prevention of further spread of *N. fulva*, especially to at‐risk areas (i.e., climatically suitable; Figs. 2 and 3), might be the best way to contain its future invasion (e.g., Bromberg et al., 2011). The information on climatic niche expansion and risk maps produced in our study can be useful tools in managing and monitoring *N. fulva* future spread and currently infested areas. For example, land managers in at‐risk areas can design policies and take appropriate steps (e.g., quarantine measures) to stop movement of *N. fulva* propagules to their regions, and thus, reduce management costs due to *N. fulva* invasion.

## Conflict of Interest

None declared.

## Supporting information


**Appendix S1.** Occurrence data, climatic variables and cross‐correlation Tables.
**Appendix S2.** Model selection summary Table.
**Appendix S3.** Variable importance and species response curves.
