# Supplementary material for: Evidence of niche shift and global invasion potential of the Tawny Crazy ant, Nylanderia fulva
Source: Ecol Evol. 2015 Sep 30;5(20):4628–41. doi: 10.1002/ece3.1737 (PMC4670064; doi:10.1002/ece3.1737)

**SUPPORTING INFORMATION**

**Appendix S1** Additional tables containing information on occurrence data, climatic variables considered in the models, and correlations among variables.

Table S1 Number of *N. fulva* occurrence points from different countries in its native and invaded range.

| Country/region | Number of occurrences | Occurrences after filtering |
| --- | --- | --- |
| Native range |  | 20 |
| Argentina | 12 |  |
| Brazil | 12 |  |
| Paraguay | 3 |  |
| Total | 27 |  |
| Invaded range (Non-CONUS) | |  |
| Colombia | 293 | 106 |
| Cuba | 4 |  |
| Dominican Republic | 1 |  |
| Ecuador (Galapagos) | 1 |  |
| Haiti | 1 |  |
| Jamaica | 4 |  |
| US Virgin Islands (St. Croix) | 6 |  |
| Total | 311 |  |
| Invaded range (CONUS) | | |
| USA (Texas) | 1032 | 181 |
| USA (Louisiana) | 3 |  |
| USA (Mississippi) | 2 |  |
| USA (Florida) | 24 |  |
| Total | 1061 |  |
| Grand total | 1399 | 307 |

**Note:**

CONUS is continental United States. Data were collected from multiple sources including Mayr (1862), Emery (1888, 1906), Ihering (1894), Forel (1908, 1913), Bruch (1914), Mann (1916), Luederwaldt (1918), Wetterer and Keularts (2008), Drees & McDonald (2014), Sharma *et al.* (2014), and Wetterer *et al.* (2014). Four species closely resemble *N. fulva* in the worker caste and thus are likely sources of misidentification. Workers of three species resemble *N. fulva* but are distinguishable based upon worker morphology: *N. guatemalensis*, *N. steinheili*, and *N. bourbonica*. A fourth, *N. pubens*, can only be reliably distinguished from *N. fulva* based on male morphology or genetic data. However, its current distribution appears to be restricted to the Caribbean region, although historical records exist from South Florida (Gotzek *et al.*, 2012). Occurrence records of the other three species in the Americas are generally restricted to Central America, the Caribbean, and Northern South America.

The type specimen of *N. fulva* was originally described from Rio de Janeiro in 1862 (Mayr, 1862). Within the contiguous region that includes, Southern Brazil (Feener *et al.*, 2008), Northeastern Argentina (LeBrun *et al.*, 2007, Calcaterra *et al.*, 2010), and Paraguay (Wild, 2007) this species is commonly reported. None of the described species that resemble *N. fulva* occur commonly in this region. Thus collection records for *N. fulva* from this region were included in the model. This region is generally considered to be the native region for this species (Fowler *et al.*, 1990; McGlynn, 1999; Fernandez, 2000), and we consider it to be so here.

Globally, three documented, regionally widespread introductions and invasions by *N. fulva* have occurred. *N. fulva* was introduced into Colombia in 1971 in two areas: Puerto Boyacá and Fusagasugá (Zenner de Polania, 1990). In Cuba, it was introduced into the province of Matanzas around 1991 (Fontenla, 1995). And it colonized the Southeastern US through Houston, Texas and Florida around 2000 (Meyers & Gold, 2008, Gotzek *et al.*, 2012). In all three of these regions, it rapidly became widespread and achieved high population densities causing economic, agricultural, or environmental problems (Zenner de Polania & Martinez, 1992; Fontenla, 1995). Other populations of unclear origin occur on islands in the Caribbean, some of which are, or have been characterized by densities sufficient to cause environmental or economic disruption (Wetterer *et al.*, 2014). Within the southern US and Colombia *N. fulva* can be distinguished from other co-occurring *Nylanderia* species by worker morphology (Fernandez, 2000; Gotzek *et al.*, 2012). However, in the Caribbean it can co-occur with *N. pubens*, making identification in the absence of males or genetic data difficult.

For consistency, within all four regions where *N. fulva* is known to occur, we included all collection records and records from the taxonomic literature that identify the species as *N. fulva*. Workers of *N. fulva* are readily distinguished from other described resident species, in the native region and in the invaded regions of the southern US and Colombia. Thus, we regard misidentification within these three regions as unlikely to bias the projected climate envelope. However, in the Caribbean, the regional co-occurrence of *N. pubens* and *N. fulva* make reliable species identification in the absence of males or genetic data difficult, increasing the likelihood of occurrence record errors from this region. However, an analysis of the climatic conditions associated with Caribbean collection records that lack males or genetic data to support them demonstrate that inclusion of these records does not alter the predicted climatic envelope of *N. fulva*. We exclude occurrence records for subspecies of *N. fulva*, as we consider the species affiliations of these entities to be unresolved. We also exclude miscellaneous, isolated collection records from the literature from regions other than the 4 described above. In the absence of many more documented occurrences in these areas, we consider these to be likely misidentifications.

**References**

Drees, B.M. & McDonald, D.L. (2014) Tawny (Rasberry) Crazy Ant; available at <http://urbanentomology.tamu.edu/ants/rasberry.html> (accessed on Feb. 4, 2015).

Sharma, S., Warner, J. & Scheffrahn, R.H. (2014) Tawny Crazy ant; available at <http://entnemdept.ufl.edu/creatures/urban/ants/tawny_crazy_ant.htm> (accessed on Feb. 4, 2015).

Bruch, C. (1914). Catálogo sistemático de los formícidos argentinos. *Revista del Museo de La Plata*, **19**, 211-234.

Calcaterra, L.A., Cuezzo, F., Cabrera, S.M. & Briano, J.A. (2010) Ground Ant Diversity (Hymenoptera: Formicidae) in the Ibera Nature Reserve, the Largest Wetland of Argentina. *Annals of the Entomological Society of America* **103**, 71-83.

Emery, C. (1988). Uber den sogenannten Kaumagen einiger Ameisen. Z. Wiss. Zool., **46**, 378-412.

Emery, C. (1906). Note sur Prenolepis vividula Nyl. et sur la classification des espèces du genre Prenolepis. *Annales de la Société Entomologique de Belgique*, **50**, 130-134.

Feener, D.H., Orr, M.R., Wackford, K.M., Longo, J.M, Benson, W.W. & Gilbert, L.E. (2008) Geographic variation in resource dominance-discovery in Brazilian ant communities. *Ecology* **89**, 1824-1836.

Fernandez, F. (2000). Notas taxonomicas sobre la "hormiga loca" (Hymenoptera: Formicidae: Paratrechina fulva) en Colombia. *Revista Colombiana de Entomologia* **26**, 145-149.

Fontenla, J.L. (1995) Un comentario sobre las "hormigas locas" (Paratrechina) cubanas, con énfasis en *P. fulva*. *Cocuyo* **2**, 6-7.

Forel, A. (1908). Fourmis de Costa-Rica, récoltées par M. Paul Biolley. *Bulletin de la Société Vaudoise des Sciences Naturelles* **44**, 35–72.

Forel, A. (1913). Fourmis d’Argentine, du Brésil, du Guatémala & de Cuba reçues de M. M. Bruch, Prof. v. Ihering, Mlle Baez, M. Peper et M. Rovereto. *Bulletin de la Société Vaudoise des Sciences Naturelles*, **49**, 203-250.

Fowler, H.G., Bernardi, J.V.E., Delabie, J.C., Forti, L.C. & Pereira-Da-Silva, V. (1990) Major Ant Problems of South-America. *Applied Myrmecology- A World Perspective* (ed. by R. Vander Meer, K. Jaffe, and A. Cedeno) pp. 3-14. Westview Press, Boulder, USA.

Gotzek, D., Brady, S.G., Kallal, R.J. & LaPolla, J.S. (2012) The importance of using multiple approaches for identifying emerging invasive species: The case of the Rasberry crazy ant in the United States. *PLoS ONE*, **7**, e45314.

Ihering, H. V. (1894). Die Ameisen von Rio Grande do Sul. Berl. Entomol. Zeitschr, **39**, 321-446.

LeBrun, E.G., Tillberg, C.V., Suarez, A.V., Folgarait, P.J., Smith, C.R. & Holway, D.A. (2007) An experimental study of competition between fire ants and Argentine ants in their native range. *Ecology*, **88**, 63-75.

Luederwaldt, H. (1918). Notas myrmecologicas. *Rev. Mus. Paul.* **10**, 29-64.

Mann, W.M. (1916). The Stanford Expedition to Brazil, 1911, with John C. Branner, Director. The ants of Brazil. *Bulletin of the Museum of Comparative Zoology*, **60**, 399-490.

Mayr, G.L. (1862) Myrmecologische Studien. *Verhandlungen der Zoologisch-Botanischen Gesellschaft in Wien* **12**, 649-776.

McGlynn, T.P. (1999) The worldwide transfer of ants: geographical distribution and ecological invasions. *Journal of Biogeography*, **26**, 535-548.

Meyers, J.M., & Gold, R.E. (2008) Identification of an exotic pest ant, *Paratrechina* sp.nr. *pubens* (Hymenoptera:Formicidae), in Texas. Sociobiology, **52,** 589-604.

Trager, J.C. (1984) A Revision of the Genus *Paratrechina* (Hymenoptera, Formicidae) of the Continental United-States. *Sociobiology*, **9**, 51-162.

Wetterer, J.K., Davis, O., and Williamson, J.R. (2014) Boom and bust of the tawny crazy ant, *Nylanderia fulva* (Hymenoptera: Formicidae), on St. Croix, US Virgin Islands. *Florida Entomologist*, **97**, 1099-1103.

Wetterer, J.K. & Keularts, J.L.W. (2008) Population explosion of the hairy crazy ant, Paratrechina pubens (Hymenoptera : Formicidae), on St. Croix, US Virgin Islands. *Florida Entomologist*, **91**, 423-427.

Wild, A.L. (2007) A catalogue of the ants of Paraguay (Hymenoptera: Formicidae). **Zootaxa**, 1-55.

Zenner de Polania, I. and O. Martinez. 1992. Impacto ecológico de la hormiga loca, Paratrechina fulva (Mayr), en el Municipio de Cimitarra (Santander). *Revista Colombiana de Entomología,* **18**, 14-22.

Table S2: Climatic variables considered in *N. fulva* niche models. General statistics were calculated using all native and invasive occurrences (n = 307); Min. is minimum, Max. is maximum, and SD is standard deviation.

| Climatic variable | Min. | Max. | Mean | SD |
| --- | --- | --- | --- | --- |
| Annual mean temperature (bio1; °C)^γ^ | 12.7 | 28.6 | 21.3 | 2.4 |
| Mean diurnal range in temperature (bio2; °C)^γ^ | 6.4 | 13.6 | 10.6 | 1.3 |
| Isothermality (bio3)^γ^ | 33 | 92 | 56 | 22 |
| Temperature seasonality (SD x 100) (bio4)^γ^ | 222 | 7089 | 3915 | 2679 |
| Maximum temperature of warmest month (bio5; °C)^γ^ | 18.0 | 36.5 | 31.7 | 3.3 |
| Minimum temperature of coldest month (bio6; °C)^γ^ | 1.5 | 23.1 | 10.0 | 5.8 |
| Temperature annual range (bio7; °C)^γ^ | 7.9 | 33.1 | 21.8 | 7.8 |
| Mean temperature of wettest quarter (bio8; °C)^γ^ | 10.4 | 28.1 | 24.7 | 3.0 |
| Mean temperature of driest quarter (bio9; °C)^γ^ | 10.9 | 28.6 | 18.4 | 4.0 |
| Mean temperature of warmest quarter (bio10; °C)^γ^ | 13.2 | 29.2 | 26.0 | 3.1 |
| Mean temperature of coldest quarter (bio11; °C)^γ^ | 9.1 | 28.0 | 15.9 | 5.2 |
| Mean annual precipitation (bio12; mm)^γ^ | 378 | 4901 | 1447 | 593 |
| Precipitation of wettest month (bio13; mm)^γ^ | 68 | 757 | 192 | 90 |
| Precipitation of driest month (bio14; mm)^γ^ | 8 | 166 | 64 | 25 |
| Precipitation seasonality (CV) (bio15)^γ^ | 7 | 70 | 32 | 12 |
| Precipitation of wettest quarter (bio16; mm)^γ^ | 192 | 2057 | 486 | 223 |
| Precipitation of driest quarter (bio17; mm)^γ^ | 44 | 574 | 225 | 82 |
| Precipitation of warmest quarter (bio18; mm)^γ^ | 157 | 946 | 398 | 157 |
| Precipitation of coldest quarter (bio19; mm)^γ^ | 44 | 1961 | 344 | 234 |
| Degree days with average temperature ≥10°C(Degdays10)^β^ | 122 | 6600 | 3949 | 906 |

Sources of data: ^γ^WorldClim (http://www.worldclim.org/; Hijmans *et al.*, 2005); ^β^Generated in ArcMap/ArcGIS software.

Reference:

Hijmans, R.J., Cameron, S.E., Parra, J.L., Jones, P.G. & Jarvis, A. (2005) Very high resolution interpolated climate surfaces for global land areas. *International Journal of Climatology*, **25**, 1965-1978.

Table S3: Pearson’s correlation coefficients (r) among climatic variables included in different models at different calibration extents; higher correlations (r ≥|0.80|) are shown in bold text. No two highly correlated variables were included in the same model.

|  | Degree days at ≥10°C | Precipitation of driest quarter (bio17) | Mean temp. of wettest quarter (bio8) | Temperature seasonality (SD * 100) (bio4) | Mean diurnal range in temperature (bio2) | Precipitation seasonality (CV) (bio15) | Precipitation of wettest quarter (bio16) | Isothermality (bio3) |
| --- | --- | --- | --- | --- | --- | --- | --- | --- |
| **Continental United States** | | | | | | | | |
| Degree days at ≥10°C | 1.00 |  |  |  | -0.11 | -0.03 | 0.18 | 0.30 |
| Precipitation of driest quarter (bio17) | 0.29 | 1.00 |  |  | -0.58 | -0.75 | 0.59 | -0.13 |
| Mean temp. of wettest quarter (bio8) | 0.59 | 0.06 | 1.00 |  | -0.11 | 0.13 | 0.04 | -0.12 |
| Temperature seasonality (SD * 100) (bio4) | -0.37 | -0.22 | 0.17 | 1.00 | -0.09 | 0.04 | -0.31 | **-0.86** |
| **NIRM-Americas** | | | | | | | | |
| Mean diurnal range in temperature (bio2) | -0.48 | -0.52 | -0.29 | 0.45 | 1.00 |  |  | -0.40 |
| Degree days at ≥10°C | 1.00 | 0.22 | **0.81** | -0.74 | -0.48 |  |  | 0.75 |
| Temperature seasonality (SD * 100) (bio4) | -0.74 | -0.19 | -0.45 | 1.00 |  |  |  | **-0.95** |
| Precipitation seasonality (CV) (bio15) | 0.20 | -0.61 | 0.21 | -0.33 | 0.28 | 1.00 |  | 0.33 |
| Precipitation of wettest quarter (bio16) | 0.71 | 0.46 | 0.48 | -0.69 | -0.58 | 0.08 | 1.00 | 0.70 |
| Precipitation of driest quarter (bio17) | 0.22 | 1.00 | 0.12 | -0.19 | -0.52 | -0.61 | 0.46 | 0.24 |
| **NIRM-Global** | | | | | | | | |
| Precipitation of driest quarter (bio17) | 0.05 | 1.00 | -0.02 | -0.25 |  |  |  |  |
| Degree days at ≥10°C | 1.00 |  | 0.79 | -0.76 |  |  |  |  |
| Isothermality (bio3) | 0.78 | 0.29 | 0.59 | **-0.91** | 0.23 | 0.21 | 0.56 | 1.00 |
| Precipitation seasonality (CV) (bio15) | 0.42 | -0.53 | 0.41 | -0.18 | 0.48 | 1.00 | 0.05 |  |
| Precipitation of wettest quarter (bio16) | 0.42 | 0.46 | 0.33 | -0.56 | -0.27 | 0.05 | 1.00 |  |
| Mean diurnal range in temperature (bio2) | 0.30 | -0.43 | 0.36 | -0.12 | 1.00 | 0.48 | -0.27 |  |

**Appendix S2** Summary of model selection.

Table S4 Summary of *N. fulva* native and invasive range model (NIRM-Americas) selection using AICc. See Table S2 for variables’ full names. Models are arranged in order of their decreasing complexity. The best model with moderate complexity is highlighted in bold.

| Variables | MaxEnt settings | No. of variables | No. of parameters | AICc | ΔAICc | Model rank |
| --- | --- | --- | --- | --- | --- | --- |
| Degdays10, bio2, bio4, bio15, bio16, bio17 | L,Q,P,T,H  (β = 1.0) | 6 | 69 | 9688.2 | -128.8 | ** |
| Degdays10, bio2, bio4, bio15, bio16, bio17 | L,Q,H (β = 2.0) | 6 | 41 | 9798.3 | -18.7 | ** |
| Degdays10, bio2, bio4, bio15, bio16, bio17 | L,Q,H (β = 3.0) | 6 | 28 | 9848.2 | 31.1 | 3 |
| Degdays10, bio2, bio4, bio15, bio16, bio17 | L,Q,P (β = 3.0) | 6 | 13 | 9820.2 | 3.1 | 2 |
| **Degdays10, bio2, bio4, bio15, bio16, bio17** | **L,Q,P (β = 2.5)** | **6** | **13** | **9817.0** | **0** | **1** |
| Degdays10, bio2, bio4, bio15, bio16 | L,Q,P (β = 2.5) | 5 | 11 | 9957.5 | 140.5 | 4 |
| Degdays10, bio2, bio4, bio15 | L,Q,P (β = 2.5) | 4 | 9 | 9973.5 | 156.5 | 5 |
| Degdays10, bio2, bio4 | L,Q,P (β = 2.5) | 3 | 6 | 10076.0 | 259.0 | 6 |
| Degdays10, bio2 | L,Q,P (β = 2.5) | 2 | 5 | 10105.6 | 288.6 | 7 |
| Degdays10 | L,Q,P (β = 2.5) | 1 | 2 | 10346.1 | 529.1 | 8 |

Note: Degdays10 is degree days with average temperature ≥10°C; L, Q, P, T and H are linear, quadratic, product, threshold and hinge features, respectively; β is regularization parameter. **These models were not ranked because the fitted response curves were very complex and did not make biological sense.

**Appendix S3** Species response curves and figures depicting relative importance of different climatic variables.

Figure S1. Relative importance of the environmental variables based on the Jackknife test. The figure shows each climatic variable’s contribution to (a) training gain, and (b) test AUC in native and invaded combined model (NIRM-Americas). Blue bars show variable importance using only that variable compared to ‘full’ model (i.e., red bar; longer the blue bar higher the variable importance), and green bars indicate how well a model performs excluding that variable.


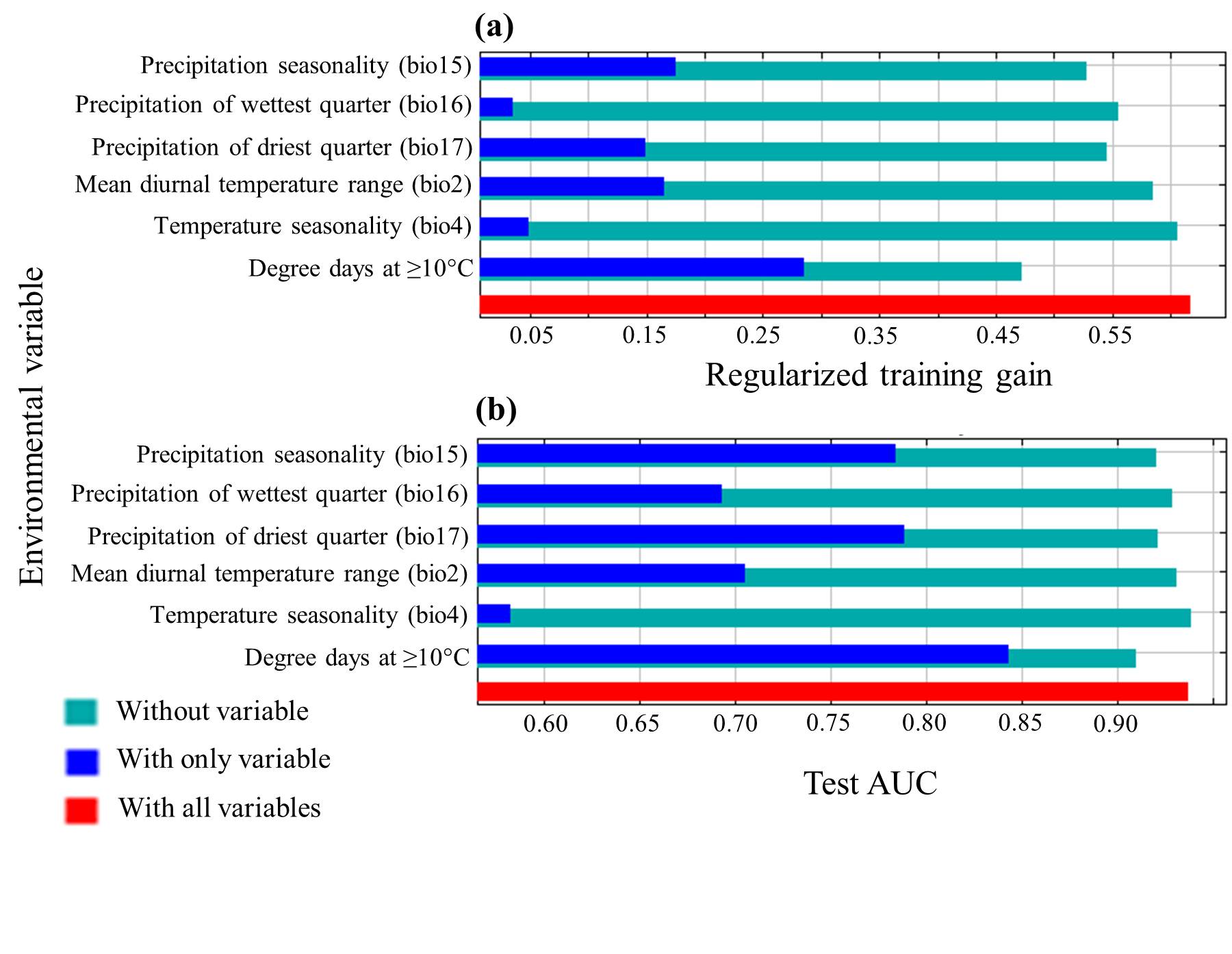


Figure S2: Response curves of the best climatic predictors of *N. fulva* in the native and invaded range combined model (NIRM-Americas); (a) mean diurnal range in temperature (bio2); (b) degree days with average temperature ≥10°C, (c) temperature seasonality (bio4), and (d) precipitation seasonality (bio15).


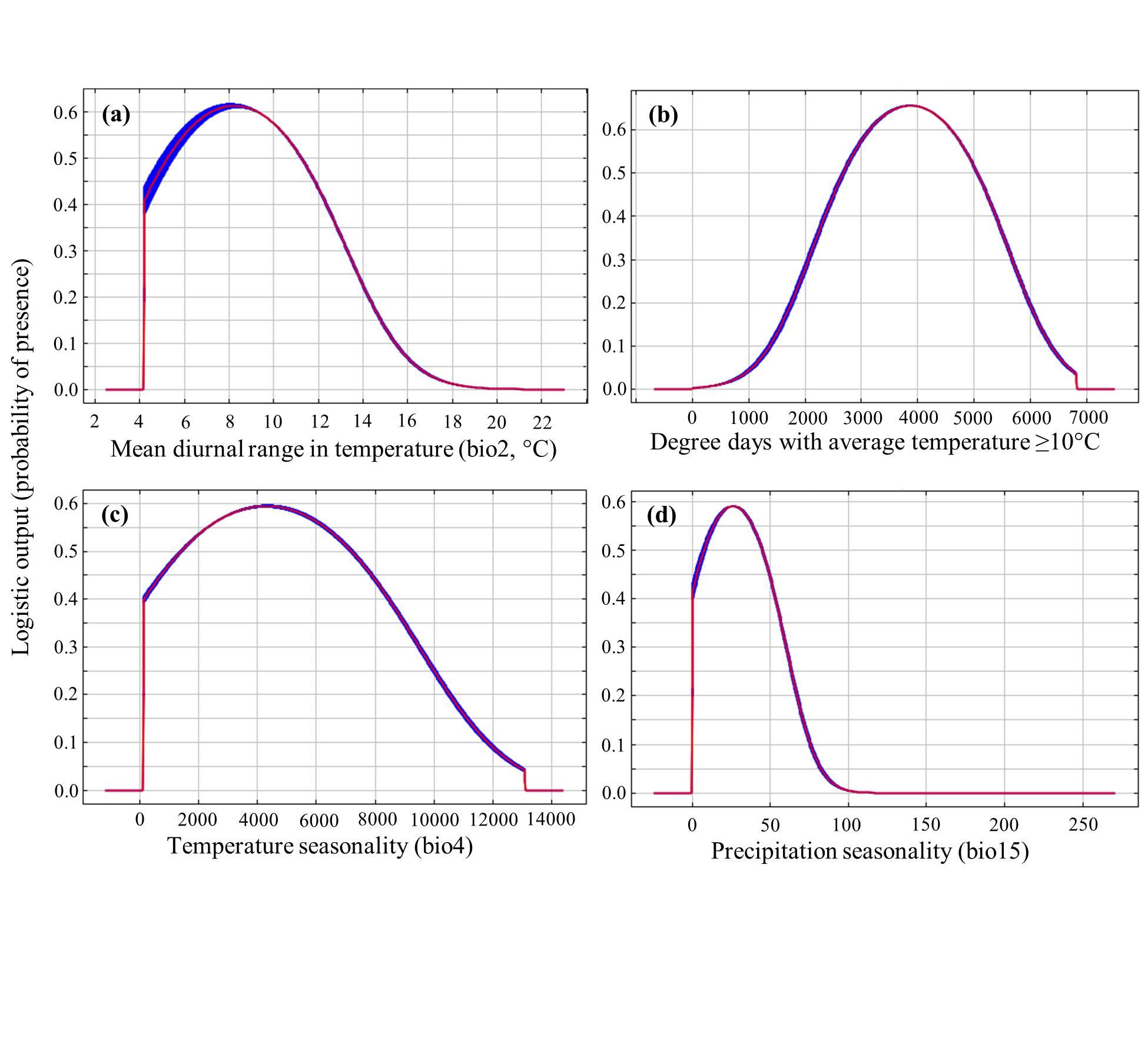

Supplement: Supplementary file 1 — Appendix S1. Occurrence data, climatic variables and cross‐correlation Tables. Appendix S2. Model selection summary Table. Appendix S3. Variable importance and species response curves. [file ECE3-5-4628-s001.docx]
